# Supplementary material for: Abrupt Gulf Stream path changes are a precursor to a collapse of the Atlantic Meridional Overturning Circulation
Source: Commun Earth Environ. 2026 Feb 26;7(1):197. doi: 10.1038/s43247-026-03309-1 (PMC12945690; doi:10.1038/s43247-026-03309-1)
Supplement: Supplementary file 2 — Supporting Information [file 43247_2026_3309_MOESM2_ESM.pdf]

Supporting Information for "Abrupt Gulf Stream path changes are a precursor to a collapse of the Atlantic Meridional Overturning Circulation"

René M. van Westen[1] and Henk A. Dijkstra[1]

[1]Institute for Marine and Atmospheric research Utrecht, Department of Physics, Utrecht University, Utrecht, the Netherlands

Contents of this file

1. Figures S1 to S8

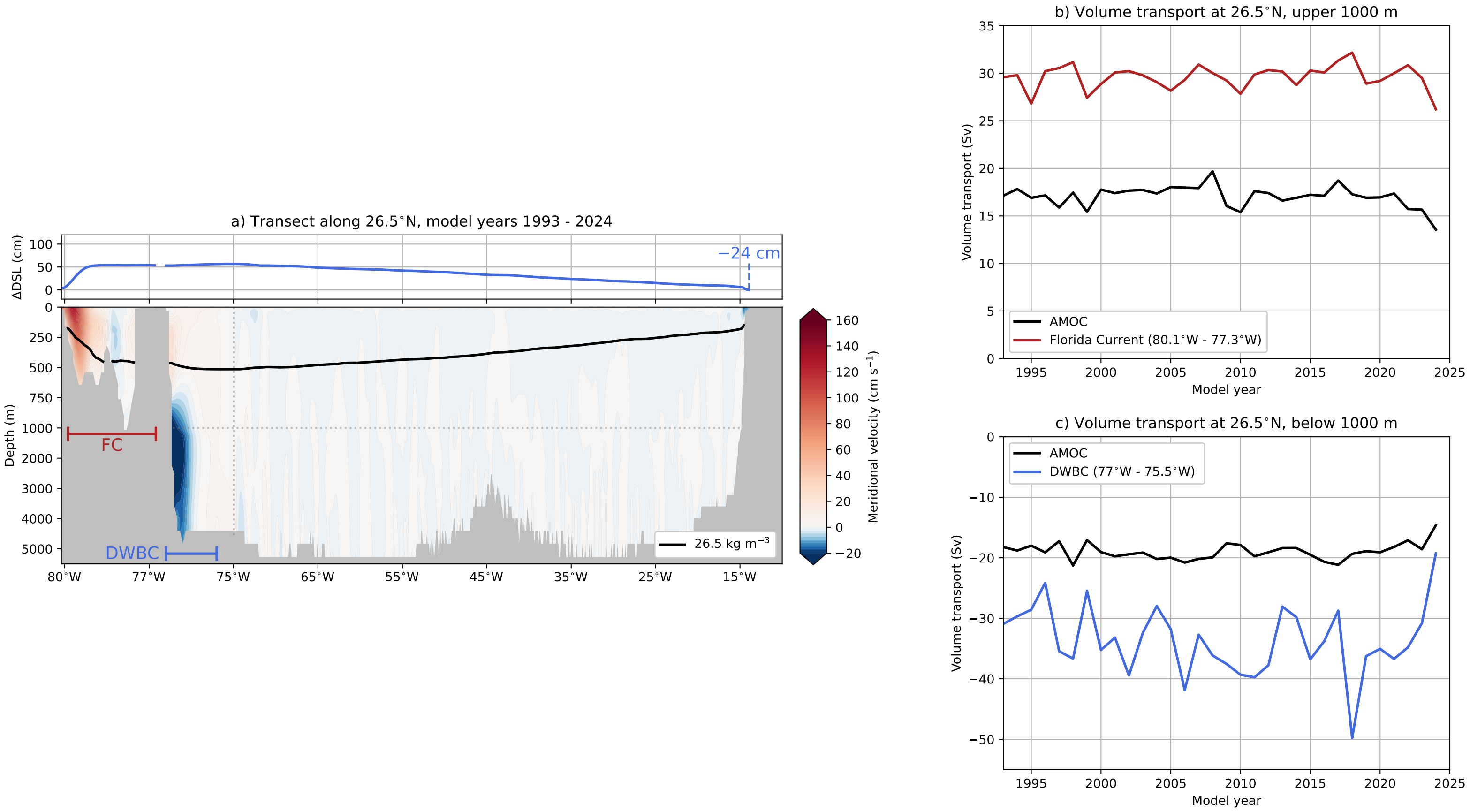

Figure S1. Oceanic Mean State along the 26.5°N transect in GLORYS12V1. Similar to Figure 1, but now for the reanalysis product GLORYS12V1 (1993 – 2024). The time-mean volume transports are 29.6 Sv (Florida Current), 17.1 Sv (AMOC, upper 1000 m), −33.6 Sv (DWBC), and −19.0 Sv (AMOC, below 1000 m).

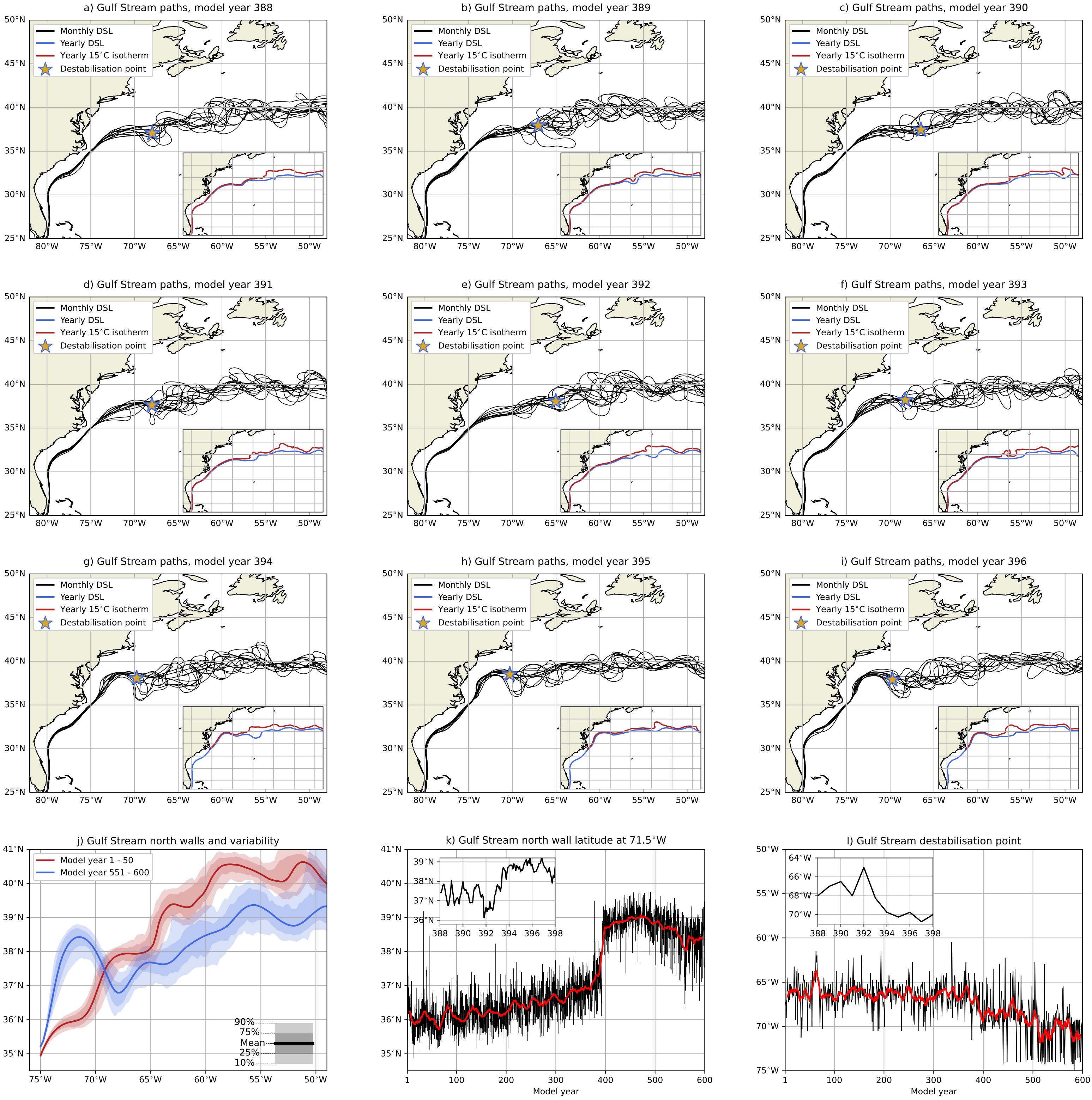

Figure S2. **Gulf Stream paths** (a – i): The monthly-averaged GS paths for model year 388 through 396, the star marker indicates the GS destabilisation point. The insets show the yearly-averaged GS paths, including the GS north wall that is based on the 15°C isotherm at 200 m depth. (j): The GS north walls for model years 1 – 50 and model years 551 – 600, comparable to Figure 2c. (k): The monthly-averaged GS north wall latitude at 71.5°W, including a 11-year moving average (red curve). The inset shows a zoomed-in version from model year 388 to 398. (l): The (yearly-averaged) GS destabilisation point, including a 11-year moving average (red curve). The inset shows a zoomed-in version from model year 388 to 398.

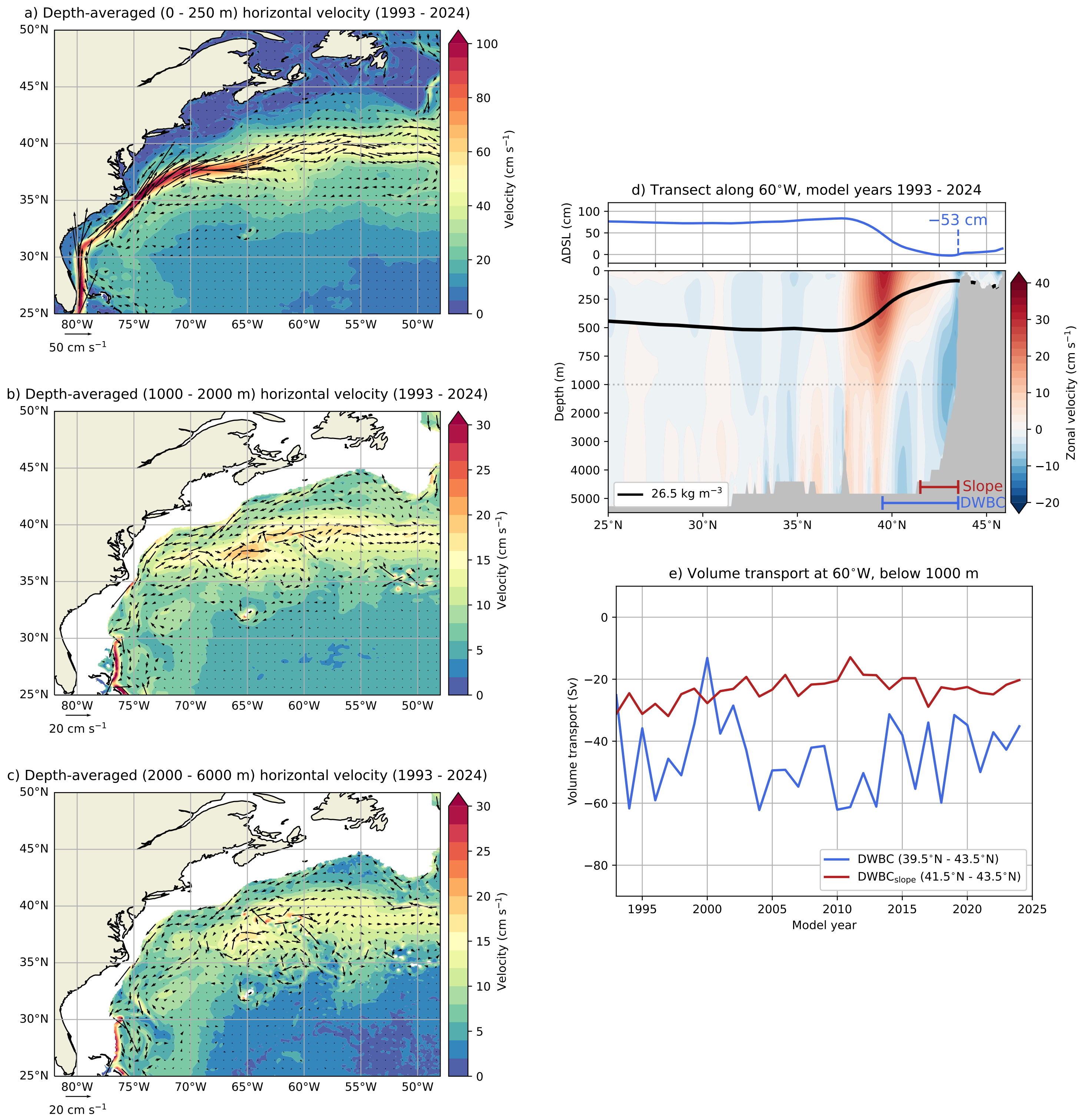

Figure S3. Oceanic Mean State over the Gulf Stream Extension in GLORYS12V1. (a – c): Similar to Figure 3, but now for the reanalysis product GLORYS12V1 (1993 – 2024). (d & e): Similar to Figure 4, but now for the reanalysis product GLORYS12V1 (1993 – 2024). The time-mean volume transports are −44.3 Sv (DWBC) and −23.3 Sv ($DWBC_{slope}$).

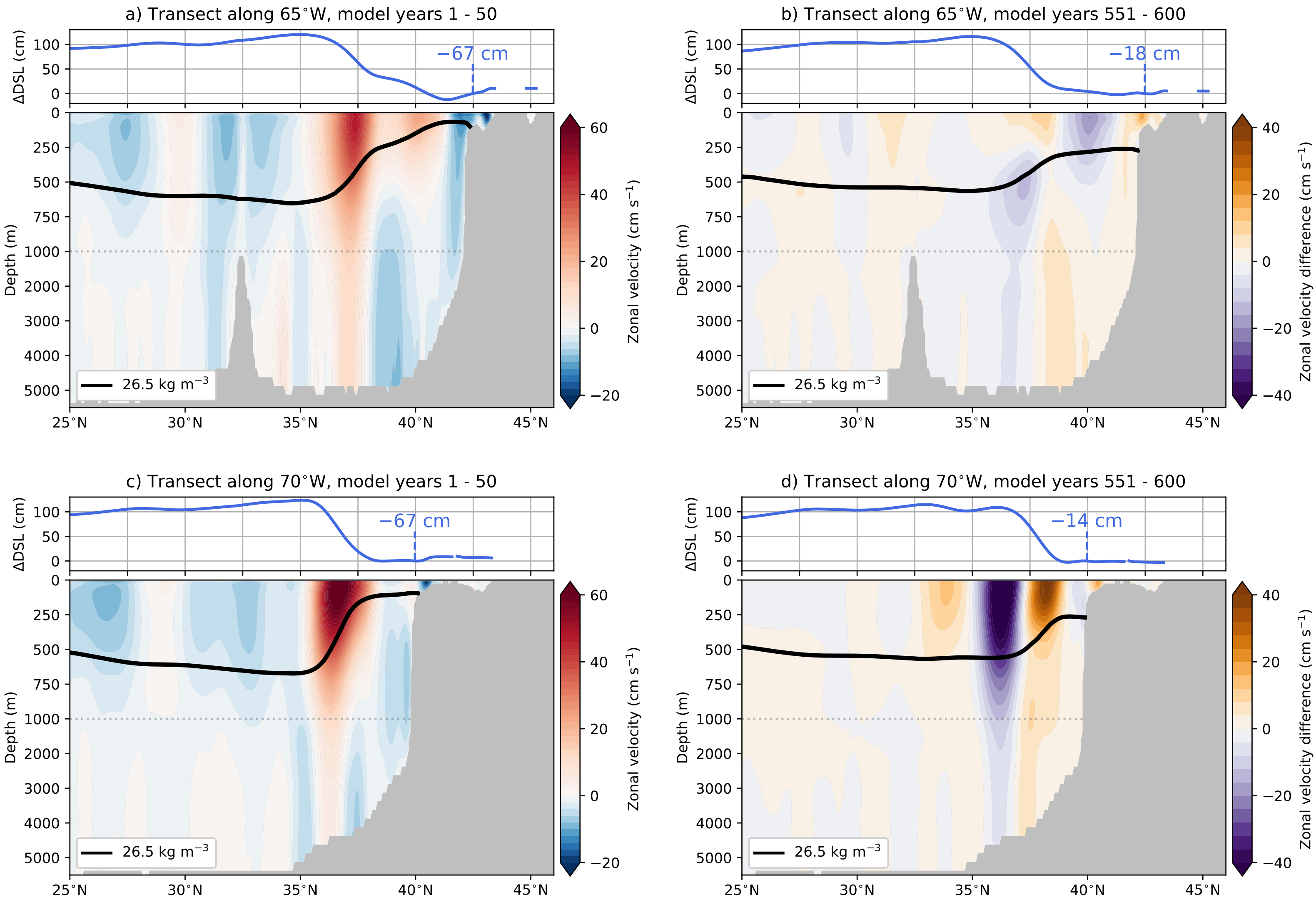

Figure S4. Oceanic Responses along 65°W and 70°W. (a & b): The DSL difference compared to 42.5°N (including its value), the zonal velocity and the depth of the $\sigma_0 = 26.5$ kg m^{-3} isopycnal along 65°W for model years 1 – 50 and 551 – 600. Only the zonal velocities for model years 551 – 600 are displayed as the differences to model years 1 – 50. Note the different spacing for the vertical axis. (c & d): Similar to panels a & b, but now along 70°W, the DSL difference is compared to 40°N. Note the slightly different ranges of the DSL differences, zonal velocity and zonal velocity difference to the ones in Figures 4a,b.

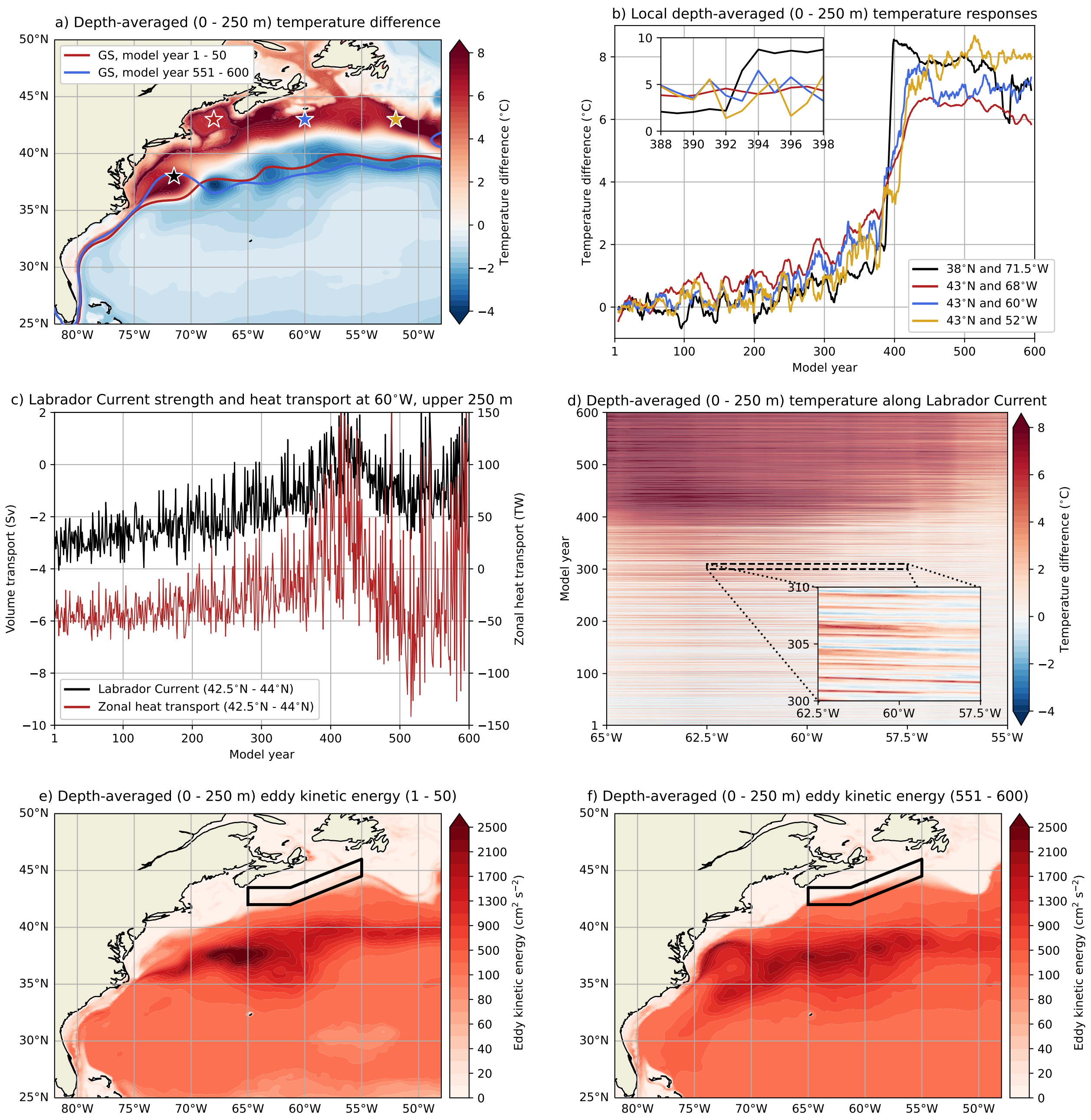

Figure S5. Temperature Responses along the Labrador Current. (a & b): The depth-averaged (upper 250 m) temperature responses between model years 551 – 600 and 1 – 50, including the GS paths. The stars indicate the local depth-averaged temperatures compared to the first 50 model years, where the yearly-averaged time series are smoothed through a 11-year moving average to reduce the variability. The inset in panel b shows a zoomed-in version for the (non-smoothed) yearly-averaged time series from model year 388 to 398. (c): The Labrador Current (42.5°N – 44°N, upper 250 m) yearly-averaged volume transport and zonal heat transport. (d): Hovmöller diagram of the depth-averaged (upper 250 m) and monthly-averaged temperatures along the Labrador Current, see black outlined region in panels e & f. The temperature difference is obtained by subtracting the climatology of the first 50 model years. (e & f): The depth-averaged (upper 250 m) eddy kinetic energy for model years 1 – 50 and 551 – 600. The eddy kinetic energy is defined as: EKE $= \frac{1}{2}\left(\overline{U^2} + \overline{V^2} - \overline{U}^2 - \overline{V}^2\right)$, where U and V are the depth-averaged zonal and meridional velocity, respectively, the bars indicate time means.

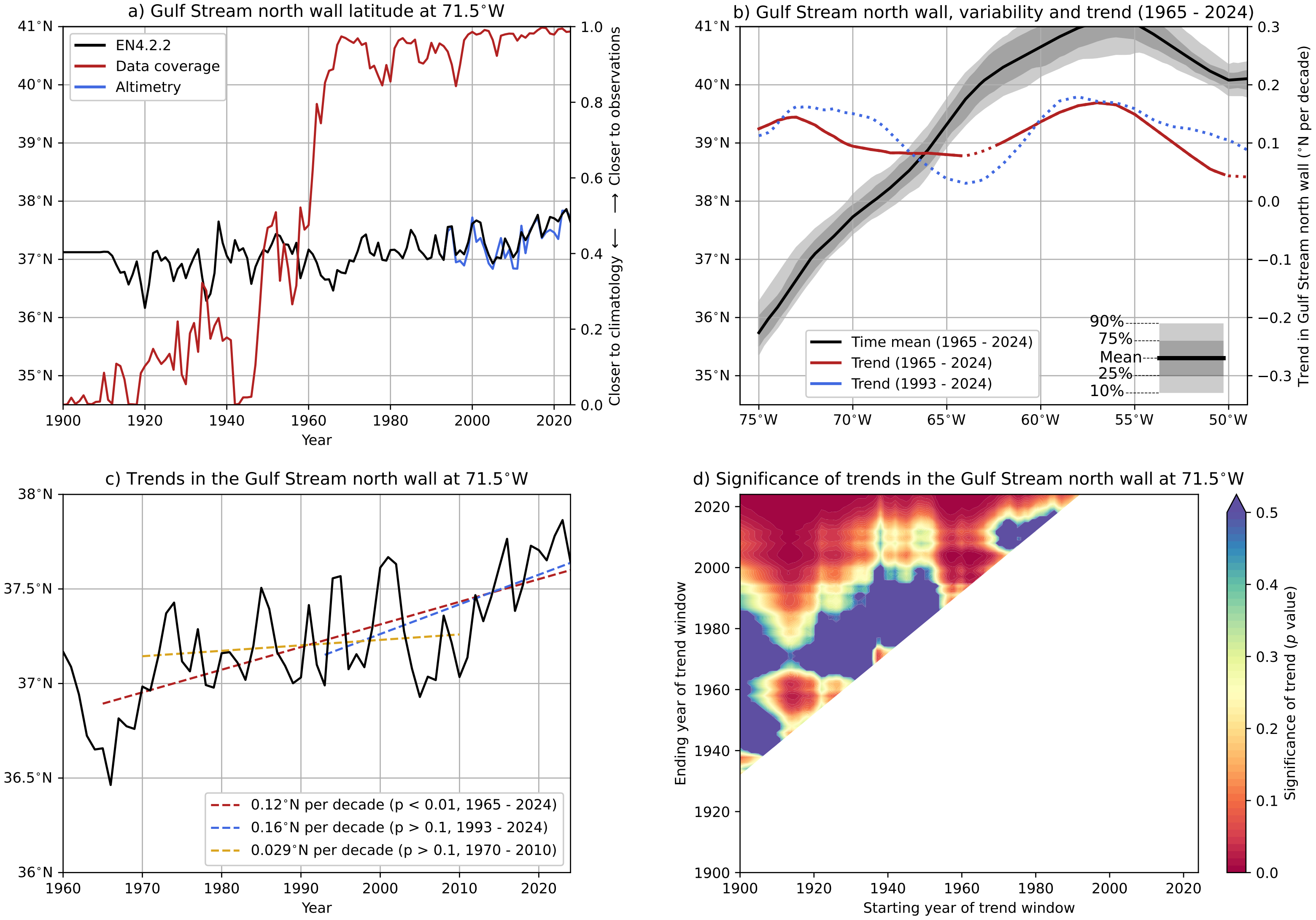

Figure S6. Gulf Stream north wall in the observational product EN4.2.2 The Gulf Stream north wall (GSNW) in the observational product EN4.2.2 over the period 1900 – 2024. For all panels, first the yearly-averaged temperature fields are obtained and subsequently the 15°C isotherm at 200 m depth is used to determine the GSNW. (a): The GSNW latitude at 71.5°W (black curve), including the data coverage (0 = no observations, 1 = observed values). The yearly-averaged GS path from altimetry is also shown (blue curve, 1993 – 2024). (b): The GSNW path, variability and trend for 1965 – 2024, comparable to Figure 6c. The GSNW trend for 1993 – 2024 is also shown. (c): The GSNW latitude at 71.5°W (black curve), including different trends (see legend). (d): Significance of trends for varying starting year and ending year, the minimum trend window is 32 years.

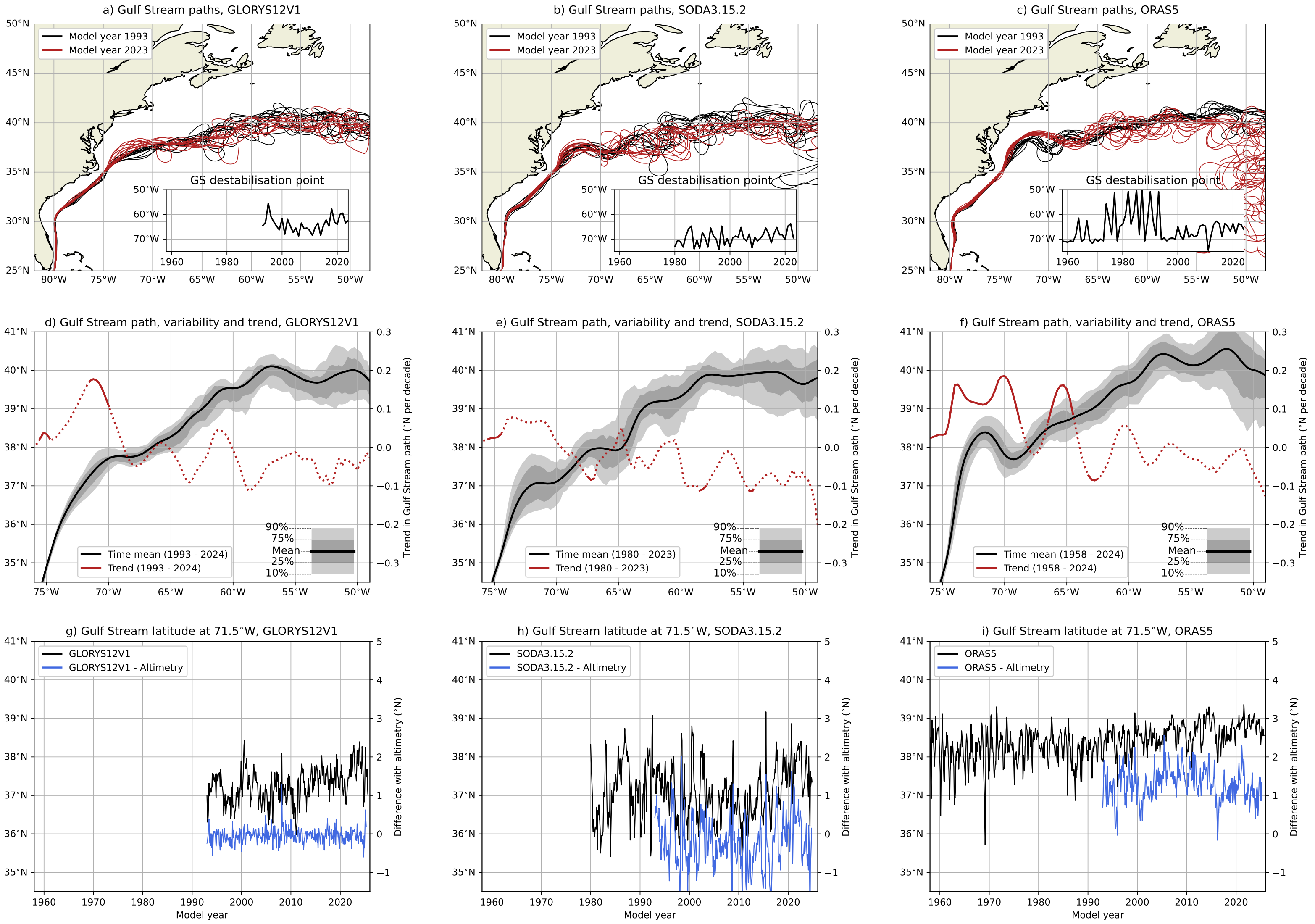

Figure S7. Gulf Stream path in reanalysis products Similar to Figures 6a,c,d, but now for the GLORYS12V1 (left column, January 1993 – August 2025, 1/12°), SODA3.15.2 (middle column, January 1980 – November 2024, 1/4°) and ORAS5 (right column, January 1958 – September 2025, 1/4°). Only entire years are considered in panel d (1993 – 2024), panel e (1980 – 2023) and panel f (1958 – 2024). In panels g,h, and i, the blue curve is the difference with satellite altimetry.

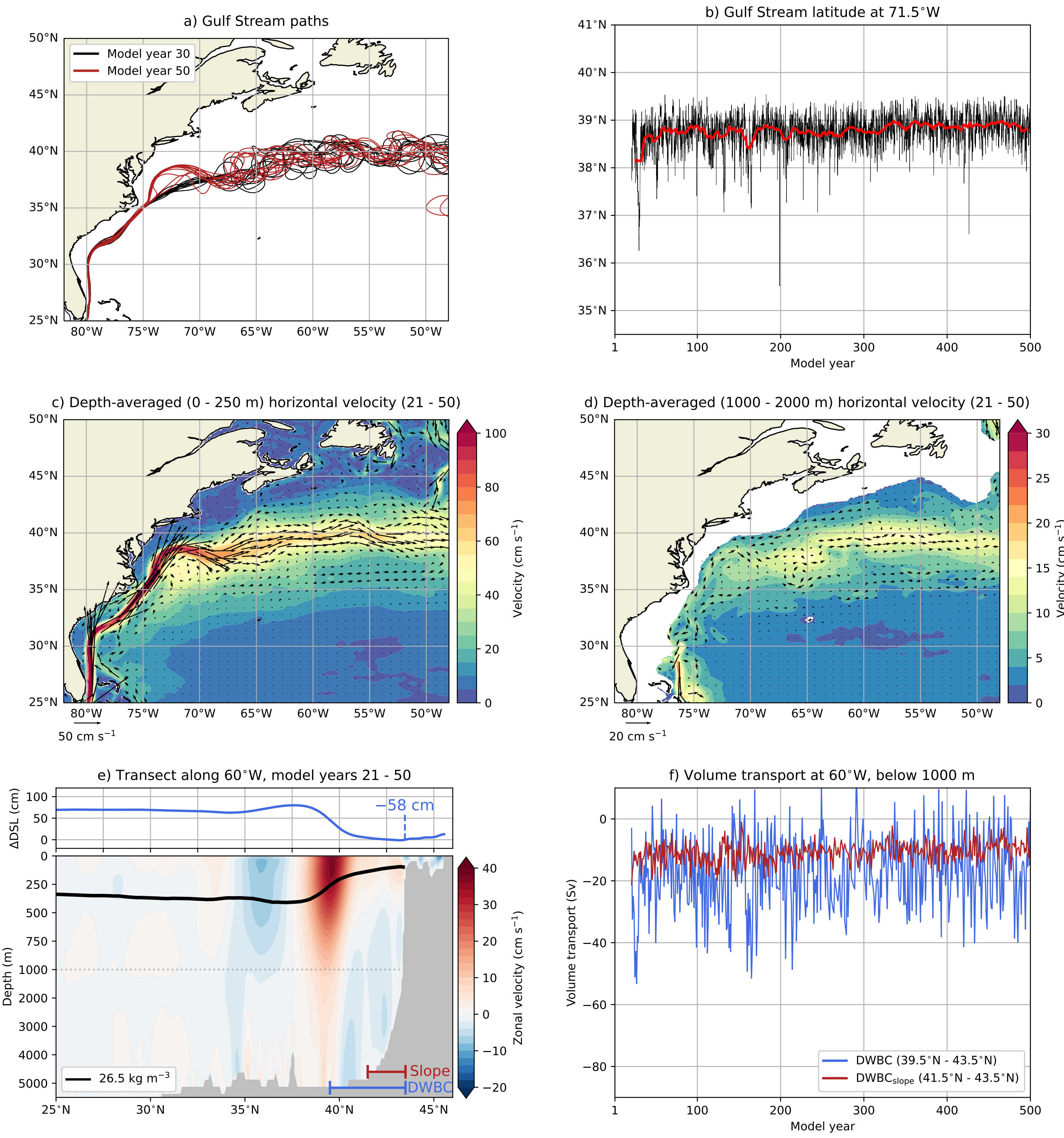

Figure S8. Gulf Stream in the high-resolution CESM Similar results as in the main text, but now for the high-resolution CESM under constant pre-industrial greenhouse gas forcing conditions. Note that the first 20 model years are not available.
